# Experiences in One Health curriculum integration and international academic collaboration: a 17-year case study in a Colombian university

**DOI:** 10.3389/fvets.2026.1951572

**Published:** 2026-10-30

**Authors:** N. Cediel-Becerra, A. Ramírez-Hernández, D. Soler-Tovar, P. Hernández-Rodríguez, P. Bermúdez-Duarte, J. F. Triana-Valenzuela, J. Regino-Vergara, A. M. Rangel-Arenas, M. Nova-Laverde, S. M. Saenz, V. Pereira-Bengoa, M. C. Corredor-Londoño, A. N. Agudelo-Suarez, E. Benavides-Ortiz, L. C. Villamil-Jimenez, D. Angulo-Mora, L. Iglesias-Hueso, J. A. Ruiz-Pinzón, E. Lefrancois, N. Bennegadi-Laurent, D. De Meneghi

**Affiliations:** 1 Epidemiology and Public Health Research Group, Faculty of Agricultural Sciences, Universidad de La Salle, Bogotá, Colombia; 2 One Health Student Club, Faculty of Agricultural Sciences, Universidad de La Salle, Bogotá, Colombia; 3 Observatorio Colombiano de Salud y Bienestar Animal OCSBA, Universidad de La Salle, Bogotá, Colombia; 4 BIOMIGEN Research Group, School of Basic and Applied Sciences, Interdisciplinary Research Incubator in Zoonotic Diseases (SIEZ), Universidad de La Salle, Bogotá, Colombia; 5 Grupo de Investigación en Medicina y Sanidad Animal, Universidad de La Salle, Bogotá, Colombia; 6 Grupo de investigación en Reproducción, Mejoramiento y Ecosistemas Agropecuarios Tropicales (REMEAT), Universidad de La Salle, Bogotá, Colombia; 7 Grupo de investigación agronegocios y sustentabilidad, Universidad de La Salle, Bogotá, Colombia; 8 Grupo de investigación Arquitectura, Experimentación y Proyecto, Taller de Vivienda Social, Semillero Estudios Rurales Campesinos y Étnicos, Universidad de La Salle, Bogotá, Colombia; 9 Grupo de investigación Economía y Desarrollo Humano, Grupo de Trabajo Ecología(s) política(s) desde el sur/Abya-Yala, CLACSO, GARN, Universidad de La Salle, Bogotá, Colombia; 10 Proyecto UTOPIA, Universidad de La Salle, Yopal, Colombia; 11 Independent Practitioner, Bogotá, Colombia; 12 IDEALISS Unit, Institut Polytechnique UniLaSalle, Rouen, France; 13 AGHYLE Unit, Institut Polytechnique UniLaSalle, Rouen, France; 14 Department of Veterinary Science, University of Turin, Turin, Italy

**Keywords:** capacity building, competencies, educational innovation, international collaboration, One Health, curriculum development, Latin America

## Abstract

This community case study documents the experience of Universidad de La Salle, Bogotá, Colombia, in progressively embedding One Health (OH) into veterinary higher education between 2009 and 2026. Responding to global calls, including those of the World Organisation for Animal Health (WOAH), for curricula that move beyond siloed, discipline-bound training, the university built a scaffolded institutional model rather than a single flagship course. The experience is organized around six complementary pillars: international academic networks and mobility; knowledge generation and translation; undergraduate research seedbeds (semilleros); interdisciplinary and veterinary OH electives; the Colombian Observatory of Animal Health and Welfare; and experiential community outreach. Together, these pillars form a multi-level strategy grounded in Colombian rural and peri-urban realities that trains undergraduate, master’s and doctoral students to work across disciplines at the human–animal–environment interface, extending OH beyond agricultural sciences into engineering, optometry and architecture. Across the pillars, international cooperation acted as a catalyst for lasting institutional change, leaving research lines, postgraduate programmes and open educational materials that outlasted external funding. Semilleros and electives proved effective, low-cost entry points: students who completed the OH elective showed a broader, more systemic understanding of the approach. Experiential learning and sequenced content strengthened competencies such as antimicrobial stewardship, while alliances around agroecology, political ecology, rights of nature and One Welfare broadened OH beyond its disease-centred focus. Persistent challenges include dependence on a few institutional champions and time-bound partnerships; limited participation of human health, environmental and social sciences; largely cross-sectional, internal evidence without systematic evaluation of learning outcomes, community impact or graduates’ practice; and weak translation of evidence into policy in a country that still lacks a national OH joint plan of action. Achieved without large dedicated funding, this experience offers resource-constrained institutions in Latin America a potential roadmap for building durable, internationally connected and locally grounded OH education ecosystems, addressing the scarcity of published models from the Latin American region. A structured curriculum assessment is proposed as the next step to benchmark and guide further integration.

## 1 Introduction

Zoonotic spillover risks and food system vulnerabilities demand a new generation of health professionals trained to integrate human, animal, and ecosystem health under the One Health (OH) paradigm (1–3). However, siloed, fragmented, and discipline-bound education can underprepare graduates to lead and participate in collaborative, intersectoral responses to the sanitary, economic, social, and ecological poly-crisis facing societies worldwide (4). The World Organisation for Animal Health (WOAH) has articulated recommendations for veterinary education that emphasize the competencies required of graduating veterinarians to address animal health, public health, and broader societal needs. WOAH also highlights the role of veterinary education institutions in preparing professionals to work across disciplinary and sectoral boundaries (5). Building individual competencies, however, is not sufficient on its own. Confronting problems as uncertain, ambiguous, and conflict-laden as those OH seeks to address calls for a different model of leadership altogether—one grounded not in individual authority but in the collective capacity of teams and networks to act under sustained pressure. Rather than continuing to invest primarily in developing single leaders, the field may need to prioritize strengthening the relational infrastructure that allows groups, institutions, and governments to act together, sustaining the collaborations required to shift entrenched systems toward outcomes that benefit health broadly and durably (4).

Competency-based training paired with collective leadership capacity—requires institutional models embedded across courses, research structures, and institutional partnerships. This translational step remains particularly underdocumented in Latin America. While global frameworks such as the One Health Joint Plan of Action (6) provide the conceptual scaffolding for OH-integrated veterinary education, the region offers comparatively few published accounts of how these principles have been operationalized in practice within university settings—through curricular design, research infrastructure (e.g., semilleros de investigación), or international academic collaboration. This scarcity limits the availability of transferable models for other Latin American institutions navigating similar structural, resource, and contextual constraints. Our research guiding question was: *how One Health was institutionalised, what enabled and constrained it, and what lessons are transferable for other institutions*. Addressing this question, the present study describes and analyzes the curricular integration and international collaborative strategies through which One Health has been embedded into veterinary higher education at the Universidad de La Salle, Bogotá (Colombia), offering a regional case study intended to inform similar efforts elsewhere in Latin America.

This case study describes a 15-year experience (2009–2026) at the Universidad de La Salle, Bogotá (Colombia) in constructing one such ecosystem. The model rests on six interconnected programmatic pillars:

1. EU-funded SAPUVETNET/ALFA network and capacity-building projects with student and faculty mobility between UniSalle, Colombia - Unito, Turin, Italy and UniLaSalle Rouen, France under Erasmus+ scholarships.
2. Knowledge Generation based on the Epidemiology and Public Health Research Group.
3. Three undergraduate research groups (“*semilleros”*) provide early scientific training at the human-animal-environment interface.
4. Three interdisciplinary elective courses operationalizing WOAH competency domains and the OH Joint Plan of Action from (WHO, FAO, WOAH, UNEP).
5. The Colombian Observatory of Animal Health and Welfare.
6. Experiential Community Outreach and extension activities: rabies preventive campaigns, AMR Capacity-Building, UNESCO and One Welfare certificate.

Taken together, these pillars constitute a deliberately scaffolded model that embeds OH competencies across undergraduate and postgraduate levels, this training model emphasizes the interdependence of human, animal, and environmental health, equipping the next generation of professionals to develop equitable, comprehensive, and resilient solutions to global health threats and leverages international collaboration to sustain curricular quality and research capacity over time (see Figure 1).

**Figure 1:**
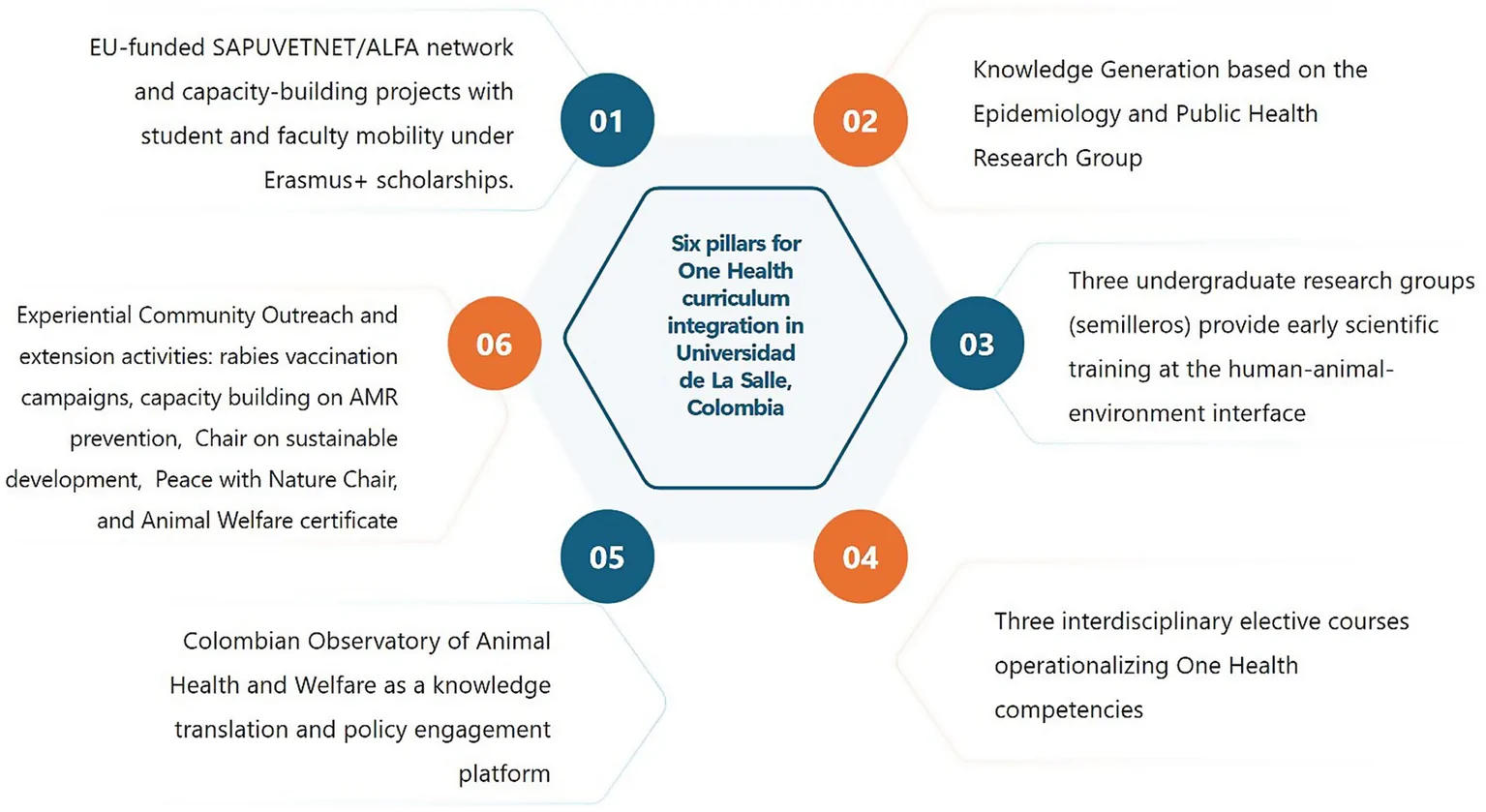
Six pillars for One Health curriculum integration in the Universidad de La Salle, Bogotá.

This case study was prepared because of its 17-year continuity and its scarcity of counterparts in Latin America. The description draws on institutional records, course syllabi, network and project reports, published outputs, internal student assessments and alumni testimonies. The information was cross-checked among co-authors from different faculties and partner institutions.

### 1.1 Positionality and reflexivity statement

Most authors are faculty directly involved in the initiatives described, which may bias the account towards achievements, a section describing lessons learned and challenges were included at the end of each pillar description in the next section.

## 2 Six pillars for One Health curriculum integration in Universidad de La Salle, Bogotá

### 2.1 First pillar: EU-funded SAPUVETNET/ALFA network and capacity-building projects with student and faculty mobility under Erasmus + scholarships

#### 2.1.1 International academic experiences and the SAPUVETNET educational legacy

The experience was anchored primarily in participation in the European Union-funded SAPUVETNET/ALFA network (2009–2012), which brought together veterinary schools from Latin America and Europe around veterinary public health (VPH) education and collaboration. This experience provided an early international framework for academic exchange, curriculum development, and collaborative work in One Health-related areas. The first SAPUVET project (2002–2004) coordinated by the Faculty of Veterinary Medicine at Utrecht University (the Netherlands), established the network’s founding premise: that VPH was largely absent from veterinary curricula across both regions, and that a harmonized, competency-based approach to teaching it was needed. A pre-existing national network of students in Colombia connected with European partners through working sessions in Buenos Aires and the Latin American Congress of Zoonoses La Plata, Argentina in 2007, brought together universities from across Europe and Latin America around a shared diagnosis: veterinary public health needed a common pedagogical language for the next generation of veterinarians. This collaboration produced a co-authored article on public health research networks in the Pan American Journal of Public Health (PAHO-WHO) (7–9), with further reflections published in 2007.

The network was reactivated through the EU’s Alfa III call as SAPUVETNET III (2008–2011), explicitly framed around contributing to the Millennium Development Goals through a One Health approach. Eleven Latin American and five European universities took part under the administrative coordination of the Universidade de Évora, Portugal, with 4 years of funding for research, mobility, and—centrally—teaching material development. The project’s opening meeting, held over a week in Bogotá, was conducted trilingually in Spanish, Portuguese, and English, setting the tone for the multilingual character of everything the network would produce (Sapuvetnet, n.d.).

Partner faculties spanned London, São Paulo, Utrecht, Turin, Buenos Aires, Baja California, Zaragoza, Havana, and Valdivia, among others, with academic direction shared collectively across the network. Between 2008 and 2012, doctoral research carried out within the network at the University of Turin generated scientific output relevant to both Colombian and Italian contexts (10, 11).

The network’s didactic output includes: a trilingual, open-access VPH/One Health teaching manual written by more than 20 professors and a harmonised curriculum proposal defining the competencies expected of veterinary graduates (12), developed with FAO’s Veterinary Public Health unit. It also produced case studies based on a problem-solving methodology, trilingual self-learning materials on meat inspection and food hygiene (76), and the trilingual journal Una Salud/One Health/Uma Saúde (13). Teaching modules were piloted through e-conferences and meetings held alternately in Europe and Latin America.

- Institutional uptake and continuity

Participation in the network catalysed concrete curricular change: new academic lines emerged, including epidemiology and public health in postgraduate programmes and food security as an undergraduate course. Recognising that the traditional organisation of the agricultural sciences could not address the complexity of tropical production systems and public health challenges, the University proposed Agrosciences as a transdisciplinary field integrating biological, veterinary, environmental, economic and social sciences to promote not only productivity but also social equity, environmental sustainability, food security and regional development (14). Within this framework, One Health was understood as part of intersectoral work towards a “new rurality,” in which agriculture fulfils multiple ecological, economic and social functions (13, 78, 81). This vision led to the creation of the master’s and doctoral programmes in Agrosciences, the consolidation of a One Health research line, subsequent international educational collaborations and the book Salud pública veterinaria: bienestar de la humanidad (15), which argued that universities should contribute to policy formulation as well as teaching and research. Collaboration among SAPUVET partners remains active, and the network’s educational materials continue to be used in VPH and One Health training well beyond its original funding period.

#### 2.1.2 Erasmus mobility funding between UNISALLE (Colombia) and UNITO (Turin, Italy) and UNILASALLE (Rouen, France)

In 2022, a framework agreement was established to formalize academic collaboration and facilitate future mobility and joint activities. Building on this agreement, academic mobility activities were implemented during 2024–2025, strengthening exchanges between the participating institutions. Subsequently, during 2025–2026, the collaboration was expanded through an Erasmus+ initiative with UniLaSalle Rouen, creating additional opportunities for academic exchange, institutional cooperation, and the development of One Health-related educational activities. The course was developed as a structured curriculum, beginning with introductory courses to establish an understanding of the OH approach.

At UnilaSalle Rouen, veterinary students receive dedicated OH instruction during their second year of study. In subsequent years, this teaching evolved into VPH education. The originality of this curriculum lies in both the number of hours specifically devoted to One Health teaching (24 h of lectures, 4.5 h of tutorials) and its sequencing, whereby veterinary public health is presented as a logical application of One Health principles.

However, the OH approach extends beyond the classroom environment. Since 2023, second-year veterinary students have participated in immersive practical learning respectively, through a two-week internship on pig or poultry farms and a one-week internship with a veterinarian or livestock advisor to see how the OH approach is being explored. In addition, for fourth-year agricultural engineering students, as part of their “Farming for the Future” career track, are offered a 25-h master class based on the soil/plant/animal/human microbiome continuum within the context of the One Health approach. These experiences allow students to apply their theoretical knowledge of the OH framework to field-based situations.

The use of the One Health Mural (“Fresque One Health”), a collaborative serious game based on collective intelligence, also contributes to the development of key competencies associated with One Health, particularly One Health literacy, critical thinking, systems thinking, and complex thinking. A tutorial session dedicated to personal values provides an opportunity for reflection and encourages students to become aware of the values they prioritize, as well as their professional and personal attitudes.

A more concise version of this program is offered to international students joining the school for a semester, as an introduction to One Health approach. Since the beginning of the veterinary curriculum, part of this One Health teaching has benefited from the experience of an international faculty member within the Erasmus program, fostering the exchange of diverse One Health perspectives and experiences.

Moreover, the OH approach has been integrated into veterinary programs and agricultural engineering curricula at different levels. Five years ago, first-year engineering students attended introductory 1.5-h lectures on animal health from a One Health approach, developing early awareness of interdisciplinary health challenges.

Based on this common conceptual approach, 50 h of specialized courses were provided focusing on emerging zoonotic diseases and antimicrobial resistance (AMR) from a One Health approach. This module emphasized the Latin American context and gender-sensitive, community-based prevention strategies. The curriculum and educational design of these courses were developed within the framework of the European Erasmus+ project LIVET—Livestock Farming and the One Health Approach in VET (2023–2026), in which UniLaSalle is a partner. The LIVET project aims to integrate the One Health approach into vocational education and training programs in the livestock sector, reinforcing the alignment between educational curricula, professional practices and public health challenges (16).

##### 2.1.2.1 Lessons learned and challenges

Participation in the SAPUVET network and Erasmus + mobility (2024–2026) among Colombia and Europe have acted as a catalyst for institutional change rather than mere academic exchange, leaving installed capacities (research lines, postgraduate programs and open educational materials) that outlasted the original funding. This suggests that the sustainability of such initiatives depends less on external resources than on their anchoring in curricular structures, and that One Health thrives where *a prior* institutional vision of transdisciplinarity, such as Agrosciences and the “new rurality,” provides the conceptual framework to sustain it. However, challenges remain between aspiration and curricular reality because the trajectory has also relied heavily on a small number of institutional champions, raising concerns about generational succession, and its impact has been documented mainly through the continued use of materials rather than through systematic evaluation of learning outcomes or changes in professional practice. Moreover, despite its transdisciplinary aims, the approach remains anchored in the agricultural and veterinary sciences, with limited participation from human health, environmental and social sciences. Finally, although the role of universities in policy formulation has been asserted, it has yet to materialize fully, and existing educational materials need updating to reflect newer frameworks, including the One Health Joint Plan of Action, global antimicrobial resistance plan of action and climate change adaptation plans.

### 2.2 Second pillar: knowledge generation and translation based on the Epidemiology and Public Health Research Group

The Epidemiology and Public Health Research Group was formed in January 1998 and currently holds an A1 classification. For more than two decades, the group has developed research and academic activities addressing epidemiological and public health challenges, including work relevant to One Health. Its vision integrates the One Health approach to connect environmental, animal, and human well-being, and it anchors the institutional One Health research agenda at the Faculty of Agricultural Sciences of the Universidad de La Salle, Bogotá. For more than two decades, the faculty has produced peer-reviewed outputs on zoonoses, antimicrobial resistance (AMR), vector-borne diseases, and EcoHealth, while supervising undergraduate, master’s, and doctoral students toward evidence-based professional practice, deeply rooted in research, education, and practical interventions addressing emerging infectious diseases, environmental health, food safety, and sustainable agriculture in the Colombian context. Methodological rigor in epidemiological analysis and innovative educational tools further strengthen the Group’s holistic OH approach—an approach with international engagement demonstrated by its participation in the final conference of the EU COST Action TD1404, Network for Evaluation of One Health (NEOH), held in Bologna, Italy, in September 2018, where the research of the Universidad de La Salle, Bogotá, presented the Latin American perspective on One Health and EcoHealth developments (presentation available online) (17).

Within this trajectory, the group’s research portfolio includes impactful epidemiological and diagnostic studies on priority zoonotic diseases. Leptospirosis has been intensively studied in animal reservoirs—including bovines, swine, rodents, canines, and chiropterans—have been considered in relation to the epidemiology of *Leptospira* spp. and leptospirosis (18–21). Complementary work has addressed the epidemiological challenges of brucellosis detection in swine production regions, highlighting persistent gaps in diagnostic capacity. Rabies, particularly of sylvatic origin transmitted by vampire bats, has also been investigated through epidemiological analyses (22–25) and evaluations of rabies vaccination programs (26), emphasizing coordinated human and animal health strategies in rural areas especially focused on domestics cats as important vectors of the disease (27) and the evaluation of national rabies programs under the OH lens (28). Besides that, systematic reviews to identify social ecological factors affecting the prevention and exposure to zoonotic diseases in Latin-American rural communities has also been conducted (29).

In the realm of vector-borne diseases, important research has been conducted. Several studies have investigated ticks, fleas and associated pathogens in various ecotones in Colombia (30, 31). These multidisciplinary investigations have assessed the ecoepidemiological and social determinants of these diseases, considering both domestic and wild animals that interact with rural communities. Particular emphasis has been placed on spotted fever rickettsioses, as they are neglected vector-borne illnesses with widespread distribution in the country and a considerable impact on public health (32–34).

Beyond disease-specific research, the group has led the production of scientific evidence of good practices for OH implementation. Examples of these are the 3 chapters in the book “Principles of One Health for a better planet” published by CABI (35) regarding Social Determinants of Health at the Human–Animal–Ecosystem Interface (36), One Health Implementation Cycle, Stakeholder Analysis and Engagement (37) and Gender Equality in the Context of One Health (38). Likewise, in the field of Antimicrobial Resistance (AMR) a key case study has been published in the OHHLEP collection of case studies about the Public, Private and Public-Private Partnerships (PPP) to Implement a One Health Approach to Combat Antimicrobial Resistance in Colombia (39, 40) and about the gap in dairy chain farmers worldwide regarding a holistic preventive approach to AMR (41). Other important lines of work have examined national OH case studies (36) and regional assessments of best practices and lessons learned across different countries in Latin America on One Health implementation (42, 43); and identifying good practices for integrating a gender perspective into One Health in the context of climate change (40, 41, 44, 45). More recently, the Group has expanded knowledge generation and translation through transdisciplinary approaches, including participatory action research, to address gender-responsive climate adaptation and the economic empowerment of rural women. This work connects household economic organization, care work, food self-provisioning and decision-making with socioecological resilience and family health and well-being. It has generated territorially grounded evidence to support gender-responsive rural extension, capacity-building and climate adaptation policies, while linking sustainable family food systems with environmental health from a One Health perspective. In this regard, impactful guidelines and frameworks to implement the OH Joint Plan of Action to accomplish the goals of equity and social inclusion have been published (46, 47).

The challenges revealed by the COVID-19 syndemic further underscored disparities in rural health access and systemic vulnerabilities (48). At the interface of wildlife and human health, the group has contributed conceptual insights on emerging infectious diseases linked to biodiversity loss and wildlife interactions, stressing the ecological importance of species such as bats and the need for conservation-sensitive disease control methods. Disaster preparedness and response involving veterinary professionals have also been advocated as a means of mitigating the public health impacts of natural catastrophes.

This trajectory is sustained by strong interdisciplinary and transdisciplinary collaboration. Diagnosis has advanced through the convergence of scientific knowledge: Biology and Veterinary Medicine have jointly contributed to understanding the biological cycles of pathogens and identifying animal reservoirs, using molecular and serological techniques to detect agents such as *Leptospira* spp. and other bacterial zoonotic pathogens, thereby facilitating early detection within the One Health framework (49, 50). Similarly, cross-disciplinary work has enabled the detection of eye infections associated with living alongside animals, promoting prevention and fostering healthier human-animal interaction (51, 52). From an ecosystem perspective, prevention and control efforts have benefited from interdisciplinary analyses of the climatic variables that determine the persistence of infectious agents in urban and rural environments (53), as well as from the identification of pathogens and development of preventive strategies that optimize animal production systems under welfare and biosafety standards, reducing the risk of pathogen transmission at the animal-human-environment interface (54). Finally, this collaborative approach has translated into tangible public health impact through two complementary avenues: strengthening epidemiological surveillance by integrating environmental and animal data into interdisciplinary predictive models and generating scientific evidence that enables government authorities to design more effective, territorially grounded control plans (55, 74).

Structural limitations identified in the group’s research include: diagnostic gaps and knowledge deficits regarding zoonotic and neglected diseases; structural inequities in rural areas and at the urban–rural interface; institutional fragmentation in health risk management; and tensions between disease control and ecological conservation. Despite significant progress in the epidemiological characterization of diseases such as leptospirosis—across domestic, wildlife, and environmental reservoirs—limitations persist regarding timely diagnostic capacity for other diseases, such as brucellosis, in agricultural production regions. Similarly, addressing emerging pathogens—such as those causing vector-borne rickettsioses—remains challenging, as these are often neglected diseases in various ecotones across the country. Analyses of emergencies like the COVID-19 syndemic have highlighted severe gaps in access to rural health services, as well as the systemic vulnerabilities and socioeconomic inequalities that hinder the effective containment of epidemiological events at the urban–rural interface. Historically, public health organization in Colombia has been fragmented, limiting the adoption of a holistic, preventive approach to cross-cutting threats such as antimicrobial resistance (AMR) within agricultural and livestock supply chains. Finally, managing wildlife-transmitted zoonoses (such as rabies transmitted by vampire bats) has underscored the difficulty of designing and implementing disease control and surveillance measures that do not compromise biodiversity conservation or ecosystem integrity.

#### 2.2.1 Lessons learned and challenges

Regarding lessons learned, key takeaways highlight the importance of a transdisciplinary and intersectoral One Health approach that integrates socioecological determinants, gender considerations and climate resilience. They also emphasize the need to translate knowledge into governance and public policy while strengthening health risk management and the veterinary role in emergency preparedness. One example of how research has informed curriculum development is the One Health elective. Its syllabus is built on the core research strengths of the group’s members, and its case studies draw on lessons learned from their projects on for example leptospirosis, sylvatic rabies and antimicrobial resistance.

Building on this experience, the group faces several challenges. As the public health funding is decreasing in the Region, the first is to move beyond disciplinary boundaries and consolidate sustainable engagement with the human-animal-environment interface through public, private and public-private partnerships in research projects. The second is to ensure that its participatory evidence, which links disease prevention and climate change adaptation to gender equity, rural women’s economic empowerment, care work and household food sovereignty, is systematically incorporated into health and environmental policy. So far the evidence generated by the group is not integrated into public policy. The third, and most pressing, is translating evidence into governance tools. Despite the group’s contributions to territorial guidelines and to proposals for national zoonoses regulations, Colombia still lacks a national One Health Joint Plan of Action and an organized university network providing coordinated technical advice on One Health; promoting both is a strategic priority. Finally, experience with health crises and natural disasters (earthquakes, floods and fires) calls for integrating epidemiological knowledge and veterinary expertise into national prevention and response systems. Although the group has been invited to consult on agricultural emergency risk management and zoonoses surveillance, prevention and control programs, it has not yet developed a training portfolio to build professional capacities in these emerging areas.

### 2.3 Third pillar: undergraduate research groups (semilleros) provide early scientific training at the human-animal-environment interface

Three undergraduate research seedbeds (*semilleros*) provide early scientific training at the human-animal-environment interface: i. the One Health student research club (within veterinary medicine), ii. the Interdisciplinary Research Incubator on Zoonotic Diseases (SIEZ), affiliated to the School of Basic and Applied Sciences), and iii. The *Semillero Ethnic Studies Student group* (within the Architecture, Urbanism and Design Faculty), whose collaboration expands OH analysis toward territorial planning and sustainable urban–rural design. Together, these pipelines instill research competencies, critical thinking, and interdisciplinary collaboration from the earliest years of undergraduate study.

- One Health Student Club (Semillero de Investigación Una Salud)

The One Health Student Club (Semillero de Investigación Una Salud) at Universidad de La Salle shows continuity as its main strength: more than a decade of sustained activity since 2013, linked to the Epidemiology and Public Health Research Group and supported by stable faculty mentorship. This has given it an institutional identity and a broad thematic agenda ranging from emerging and neglected zoonoses to conservation medicine, disasters, food security and animal health economics. The student club has enabled undergraduate students to produce publishable knowledge, such as the prioritization of viral zoonoses at the wild pig-domestic animal-human interface published by Colombia’s National Institute of Health, a prospective evaluation of zoonosis initiatives in Latin America, to engage with urban and rural communities, and to participate in national and international networks and events such as the One Health Colombia International Symposium, the International Student One Health Alliance and One Health Day. In this sense, this *semillero* serves as an early training space in which One Health becomes research practice, science communication and engagement with health authorities, rather than a merely declarative curricular content. Remarkably, one graduate from this group participated in the Rabies in the Americas Conference RITA 2023, and was selected as the best oral presentation of the young presenters of the conference.

#### 2.3.1 Lessons learned and challenges

This *semillero* participated in the *Una Salud en Colombia* book (2025) and reported eight conference participations and 12 publications, which is modest for a decade of activity. As a group we have not yet made an assessment of learning outcomes, changes in competencies or actual influence on policy decisions; our contribution to decision-making is asserted but not documented with a valid and transparent methodology. The student club remains essentially veterinary, with limited participation of students from human health, environmental or social sciences, which strains its own transdisciplinary aims, and its trajectory depends heavily on faculty leaders, raising concerns about succession.

The lessons that emerge are that this research student group is an effective, low-cost entry point for institutionalizing One Health in university education; that sustainability is achieved when they are anchored in a consolidated research group; and that their next step should be to open up to other disciplines and universities, adopt mixed and participatory methods with impact evaluation, and explicitly link their outputs to governance instruments, contributing to the university network that Colombia still needs to provide coordinated One Health advice.

- Interdisciplinary Research Incubator in Zoonotic Diseases (SIEZ) of the School of Basic and Applied Sciences

This interdisciplinary research incubator involves students from academic programs in Biology, Veterinary Medicine, Animal Science, Optometry, and Environmental Engineering of the Universidad de La Salle in Bogotá. The research incubator is affiliated with the BIOMIGEN Research Group of the School of Basic and Applied Sciences and seeks to share knowledge based on research in human and animal health in the context of “One Health.” It works primarily on the study, diagnosis, prevention, and control of diseases with public health implications. With a 12-year track record, the group has involved diverse members who, over time, have achieved: publication of national and international articles; participation in events; grants through calls for Young Researchers; add distinctions and awards. Recent studies published by undergraduate students have revealed the effect of curcumin, reserpine, and their combinations with two commercial antibiotics (gentamicin and azithromycin) on the QS mechanisms of *Pseudomonas aeruginosa*: the expression of the *mvfR* gene and the production of pyocyanin and rhamnolipids. The results conclude, the mixtures of curcumin and gentamicin and reserpine and gentamicin may become alternatives to complement or enhance conventional methods currently used to treat *P. aeruginosa* infections, a pathogen of critical priority, according to the WHO (56).

In the current epidemiological landscape, where nearly 75% of emerging infectious diseases are of zoonotic origin (1, 6) the involvement of students in training—at the undergraduate, master’s, and doctoral levels—in the fields of veterinary science, basic sciences, and health sciences is a vital component of the One Health framework, enabling a shift from the traditional clinical perspective toward the One Health paradigm. Through their work dynamic, SIEZ students have received training from ULS faculty members as well as national and international visiting professors through the course “Geographic Information Sciences for Health Applications” (2018), taught by a researcher from the University of Illinois and organization an event —the Symposium on Research in Epidemiology and Molecular Pathogenesis of Tropical Diseases (2016). Renowned researchers from national universities such as the University of Antioquia, the National Institute of Immunology, and the National Institute of Health participated in this event. Additionally, alongside the symposium, a course titled “Bioinformatics Workshop for the Analysis of Bacterial Genomes” was offered. This course was led by two international researchers from the Center for Genomics and Proteomics at Monash University and the Center for Computer Science at the University of Melbourne (Australia).

- Collaboration with the Social Housing Workshop articulated to the research project Vulnerable Rural Territories and the Rural Peasant and Ethnic Studies Student group (Faculty of Architecture, Design and Urbanism of the Universidad de La Salle, Bogotá)

The articulation between the Workshop on Social Housing in the Villa of San Benito Abad (Sucre, Colombia), the research focus One Health and the academic spaces of the Faculty of Agricultural Sciences of the Universidad de La Salle, Bogotá, offers a privileged terrain to consolidate research, training and social projection processes in which architecture, agricultural sciences and environmental health converge. This alliance makes it possible to address rurality not only as an area of material shortages or exposure to climate threats, but as a complex socio-ecological system, in which housing, family production, water, animals, health and community practices are closely related. This perspective is consistent with the understanding of rural habitat as a web of relationships. Coronado Ruiz (57) points out that rural habitat corresponds to a conceptualization that considers phenomena “of social, cultural, ecological, environmental order,” and that seeks to understand not only the objects or subjects that shape a territory, but also “the intermingled relations between them” (p. 109). From this approach, rural housing cannot be understood as an isolated unit or as a strictly constructive object, but as part of a territorial system where habits, productive practices, family relationships materialized, environmental dynamics and cultural ways of inhabiting.

In this sense, the One Health approach broadens the architectural reading of the territory by allowing us to understand housing as an interface between human health, animal health and ecosystem health. In San Benito Abad, the location of corrals, contact with animals, water quality and management, waste disposal, food production, Basic sanitation and ventilation conditions of domestic spaces are variables that simultaneously affect habitability, family well-being, rural productivity and prevention of health risks. For this reason, the proposals of adaptive housing must go beyond a purely physical-spatial response and constitute strategies of care, prevention, adaptation and territorial resilience. The relevance of this approach is reinforced in flood-prone territories. Rangel Arenas et al. (58) warn that “the issue of natural and socio-natural disasters” constitutes a central issue for research, since it affects “the preservation of the human being and his environment” (p. 8). This assertion allows for the recognition that risk does not depend solely on the occurrence of a natural phenomenon, but on the social, spatial, environmental and productive conditions that increase or reduce the vulnerability of communities.

Therefore, working in San Benito Abad from the articulation between architecture, agricultural sciences and One Health requires considering that floods are not only events that affect physical infrastructure, but also transform everyday practices, alter mobility, modify the relationship between housing and production, affect water quality, increase pollution risks and may promote adverse conditions for human and animal health. This idea allows to orient the academic work towards adaptive solutions that recognize water not only as a threat, but also as a structuring element of life, economy, culture and territorial organization.

The experience of the Social Housing Workshop offers a relevant academic scenario for developing this articulation. According to Rangel Arenas (59), the Workshop “analyzes the habitat at its different scales” and recognizes “the importance of the habitat’s quality of life” for territorial development (p. 3). This orientation allows students to understand that housing design is not limited to solving the internal distribution of a building, but involves relationships with equipment, productive spaces, infrastructure, basic services, landscape, water systems and living conditions of communities. Likewise, the syllabus of the Workshop states that students must develop the ability to propose housing that “positively impact the territory, generating a dignified and comfortable habitat” (59, p. 3). In this framework, the incorporation of the One Health approach allows to expand the notion of positive impact: decent and comfortable housing must be healthy, safe, adaptive, environmentally responsible and consistent with the productive dynamics and culture of rural families.

From this perspective, the joint work between students of architecture, engineering, veterinary medicine, animal husbandry and agricultural sciences can guide comprehensive diagnoses on conditions of habitability, zoonotic risks, animal welfare, food safety, environmental quality and vulnerability to flooding. The value of this articulation is that epidemiological, environmental and socio-spatial data can inform design decisions. Thus, aspects such as the location of corrals, drainage conditions, water management, separation between domestic and productive areas, waste disposal, food protection and ventilation of spaces become design criteria for health and habitat.

The interdisciplinary nature of experience should not be understood as a sum of isolated knowledge, but as a condition for interpreting rural territories integrally. The Workshop on Social Housing discussion paper argues that “the complexity of these environments requires a multidisciplinary approach that integrates diverse knowledge and perspectives” to address the social, economic, cultural, environmental and productive dimensions of habitat (60, p. 5). This statement is particularly relevant for the articulation with One Health, as it allows architectural design with public health, veterinary medicine, agricultural production, environmental management and community participation. In the same sense, Rangel Arenas (60) argues that architecture in rural contexts should not be assumed as an end product or a technical response disconnected from social processes. The author argues that design should be understood as an educational and participatory practice that articulates technical, cultural and experiential knowledge, and promotes contextualized, sustainable and culturally relevant solutions. This conception allows the housing project to be placed within a collective learning process, in which students, teachers, professionals, rural families and local institutions produce shared readings about the territory and build transformation alternatives.

##### 2.3.1.1 Evaluation and impacts

The evaluation of experience should assess both the training results and the territorial relevance of the processes developed. For this, it is recommended to combine quantitative and qualitative tools that allow understanding changes in student learning, the quality of interdisciplinary articulation, community participation and relevance of urban-architectural proposals.

Rangel Arenas (60) proposes to evaluate meaningful learning through two moments: an “entry point” and an “exit point,” applied at the beginning and end of the academic term. According to the author, this resource seeks to evaluate the evolution of students’ responses to open and closed questions, especially in relation to the quality of their critical and reflective discourse. This strategy can be adapted to the One Health experience to identify transformations in territory understanding, integration of health and environmental variables, projectual argumentation and interdisciplinary working capacity.

The findings of the previous experience of the Social Housing Workshop constitute a useful reference for this evaluation. Rangel Arenas (60) reports that 93% of students developed disciplinary competencies in architectural design adapted to the territory and skills associated with service-learning. In addition, 78% said that they had reached a high level of understanding of the realities and complexities of rural territories of La Mojana through direct contact with the community, while 22% indicated that they had reached a good level of understanding. These data should not be automatically generalized to other academic groups, but they do provide a baseline for formulating goals and indicators in future interdisciplinary experiments.

The same evaluation reported that 100% of the students considered that the Workshop allowed to establish a close and significant relationship between academic learning, the realities of the rural territories of La Mojana and the community; also, 100% recognized that the space favored reflection from different forms of knowledge and the development of adaptive alternatives linked to peasant identity (60, pp. 17–18). These results suggest that integration between teaching, territory and community participation can strengthen vocational training and university social responsibility. As can be seen in Figure 2.

**Figure 2:**
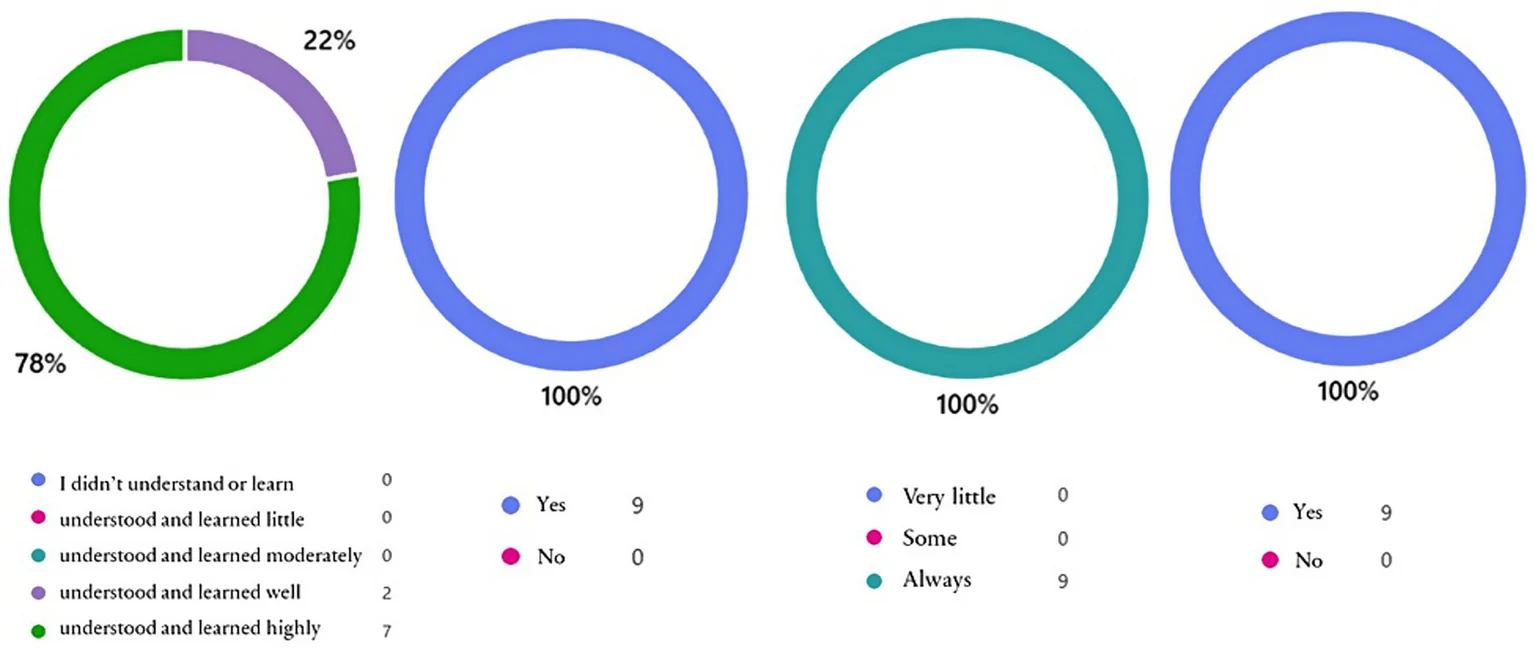
Results of evaluation of students on the Workshop of Social Housing (FADU) of the Universidad de La Salle. Source: Own elaboration 2024–2025, Adapted from Rangel Arenas (60), p. 18, licensed under CC-BY 4.0.

In conclusion, the articulation between the Workshop on Social Housing, the One Health incubator and academic agricultural science spaces can consolidate a training and research ecosystem of high value for Colombian rurality. His main contribution consists in understanding housing not as an isolated artifact, but as part of a network of relationships between people, animals, water, production, health, environment and culture. From this perspective, the evaluation should not limit itself to rating project products but should value students’ ability to interpret complex socioecological systems, work with other knowledge, build relevant proposals and participate in processes of territorial transformation with the communities.

### 2.4 Fourth pillar: four interdisciplinary-disciplinary elective courses

Interdisciplinary and Veterinary One Health Electives (2004–2026): The integration of One Health into the Veterinary Medicine curriculum of Universidad de La Salle evolved in three stages between 2004 and 2026 (Figure 3). First, Conservation Medicine courses (2004–2006; 2013–2016) introduced ecological, veterinary, epidemiological, and social perspectives that anticipated the One Health and One Welfare paradigms. Second, Wildlife Management and Wildlife Medicine broadened this foundation by linking biodiversity conservation, ecosystem health, animal welfare, and public health. Third, since 2018, three complementary curricular innovations—an interdisciplinary One Health & Sustainable Development Goals elective (currently not offered), a *Peace with nature* elective and a veterinary *One Health* elective—have consolidated structured, competency-based training aligned with the One Health High-Level Expert Panel (OHHLEP) definition and the One Health Joint Plan of Action. Across these stages, authentic case studies, expert seminars, collaborative discussions, and science-communication activities have progressively strengthened the 5 one health principles, systems thinking, epidemiological reasoning, interdisciplinary collaboration, and evidence-informed decision-making at the human–animal–environment interface.

**Figure 3:**
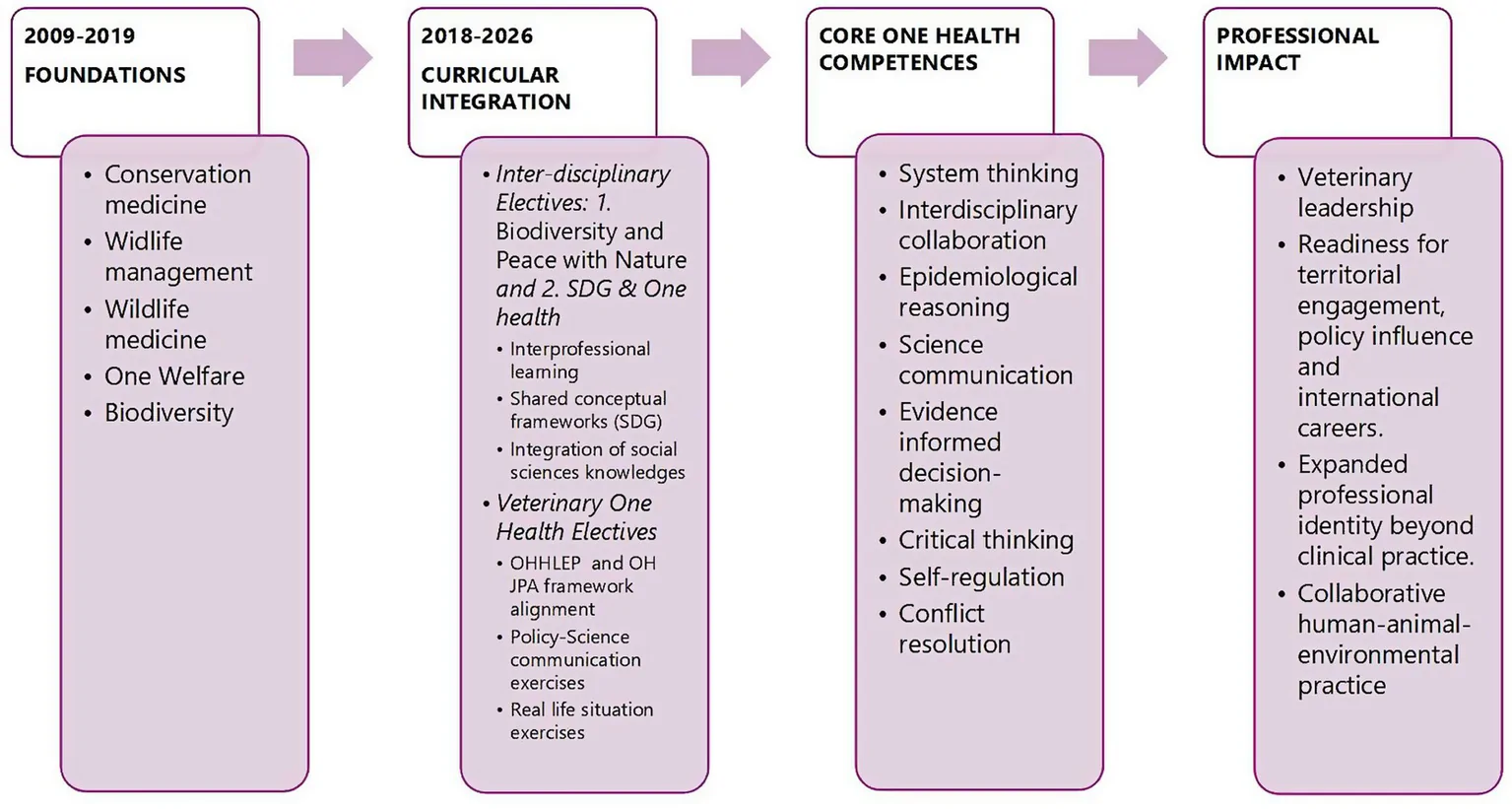
Evolution of One Health curriculum integration at the Universidad de La Salle, Bogotá (2004–2026).

In the context of the sixteenth meeting of the Conference of the Parties to the Convention on Biological Diversity (COP16), held in Cali, Colombia, from 21 October to 1 November 2024, the University introduced the interdisciplinary elective course “Biodiversity and Peace with Nature” in the second semester of 2024. The course facilitated dialogue among students from various degree programmes, faculty members in Economics, Veterinary Medicine, Basic Sciences, Habitat Sciences, and Education, and grassroots organisations of women environmental defenders.

The course critically examined the narratives and geopolitical configurations underpinning the United Nations’ approach to “sustainable development,” bringing them into dialogue with the integral ecology advocated by Pope Francis and the rights of nature championed by the Global Alliance for the Rights of Nature (GARN). Building on this conceptual foundation, the exploration of the meanings of, and pathways towards, “Peace with Nature” incorporated One Health alongside agroecology, bioeconomics, the rights of nature, and critical environmental education.

This process culminated in the Manifesto for Socio-Environmental Justice and Peace with Nature, developed by students through a participatory process within the academic community. Students presented the manifesto in the COP16 Blue Zone, and it remains subject to ongoing review in subsequent iterations of the course, which continues to be offered. The central challenge emerging from this experience concerns the demand from students in more recent cohorts to translate the manifesto’s declarations into a more concrete programme of institutional action (61).

Figure 3 makes this curricular progression explicit. The first stage (2004–2016) established an ecological and population-health foundation through Conservation Medicine. The second stage broadened that foundation through Wildlife Management and Wildlife Medicine, integrating biodiversity, ecosystem health, animal welfare, and public health. Since 2018, the third stage has consolidated One Health through complementary interdisciplinary and veterinary electives, moving from exposure to human–animal–environment connections toward structured, competency-based training.

During March and April, 2026, a cross-sectional survey was administered to Veterinary Medicine students at Universidad de La Salle, and responses were analysed collaboratively by students enrolled in the course as a formative research exercise. The objective was to explore the contribution of the One Health elective to students’ understanding of the One health approach. Of the 44 respondents, 13 (29.5%) had completed the elective and 31 (70.5%) had not. Most participants were women (79.5%), consistent with the ongoing feminisation of the veterinary profession, and more than half were in their third semester (54.5%), with smaller representation from the sixth to ninth semesters. The questionnaire combined closed and open-ended items addressing familiarity with the One Health approach, its definition, perceived relevance for disease prevention and surveillance, professional advantages, the veterinarian’s fields of action, and recognition of the “4 Cs” (communication, collaboration, coordination and capacity building).

All students who had completed the elective reported knowing what the One Health approach is and what it is for, and their definitions consistently integrated human, animal and environmental health, their interdependence, and the need for prevention. Among those who had not taken the course, 41.9% reported knowing the approach, 29.0% had heard of it without a clear understanding, and 29.0% did not know it; their definitions tended to be partial or limited to animal health and disease prevention, frequently omitting the environmental dimension. Nevertheless, the perceived importance of One Health for disease prevention, surveillance and control was high across both groups: nearly all students who completed the elective gave it the highest rating, while the only two neutral responses came from students who had not taken it. This suggests that the concept already permeates other curricular spaces, while the elective consolidates it into structured, operational knowledge.

Differences were also observed in the depth with which students articulated the professional value of the approach. Those who completed the elective most frequently cited zoonosis prevention (8), followed by interdisciplinary collaboration (4), antimicrobial resistance (AMR) (3), and employment opportunities or added professional value (3), and they identified public health and research as key fields of action, together with biosecurity, epidemiological surveillance, environmental management and wildlife health. Students who had not taken the course also identified zoonosis prevention and public health as the main advantage (15), but their responses were more general and oriented towards clinical practice, food safety and animal welfare, with AMR mentioned only once. Similarly, when asked about problems in which One Health is relevant, both groups mentioned emerging zoonoses, AMR and environmental contamination, but only students who had completed the elective extended their answers to biodiversity loss, ecosystem fragmentation and food security. Overall, these findings indicate that the elective is associated with a broader, more systemic and professionally oriented understanding of One Health, shifting the perception of the veterinarian from a predominantly clinical role towards strategic functions in surveillance, AMR control and ecosystem health. However, the results should be interpreted as exploratory, given the small sample, the unequal group sizes, the absence of inferential statistical analysis and the fact that exposure to the elective overlaps with a more advanced stage of training. Even so, they support the recommendation to introduce the One Health approach earlier and transversally across the Veterinary Medicine curriculum and other academic programmes.

#### 2.4.1 Lessons learned and challenges

The conservation medicine and wildlife management electives demonstrated that interdisciplinary and One Health-oriented learning is strengthened when conservation challenges are addressed through situated, problem-based educational experiences. Unfortunately, conservation medicine electives are not currently offered in the veterinary curriculum. However, the Elective on One health and the one Peace with nature are still being offered during the last 2 years. Bringing together students from different disciplinary backgrounds created opportunities to connect biodiversity conservation with animal, human, and ecosystem health, while encouraging students to move beyond discipline-specific perspectives toward systems thinking and collaborative problem-solving.

The survey carried out as a class exercise was designed, applied and analysed by students enrolled in the elective, who thereby moved from being recipients of One Health content to generating evidence about its place in their own curriculum. In their reflections, the student teams highlighted the development of skills in data collection, organisation, critical analysis and interpretation, as well as the value of listening to the academic community as a basis for continuous curricular improvement.

Key challenge has been sustaining interdisciplinary engagement across students with different disciplinary backgrounds, levels of prior knowledge, and academic expectations. Ensuring meaningful participation and maintaining a common One Health framework requires continuous adaptation of teaching strategies, collaborative activities, and conservation cases so that students can contribute from their respective disciplines while developing a shared understanding of complex socioecological problems.

### 2.5 Fifth pillar: the Colombian Observatory of Animal Health and Welfare as a knowledge translation and policy engagement platform

Universidad de La Salle established the Colombian Observatory of Animal Health and Welfare (OCSBA) in 2021 as a permanent platform for knowledge translation, academic dialogue, data analysis, and policy engagement within a One Health framework. The Observatory connects scientific evidence, professional training, public discussion, and decision-making around issues at the human–animal–environment interface. In this way, it complements curricular innovations, research seedbeds, postgraduate training, community-based activities, and international collaboration by extending One Health work beyond formal teaching spaces (75).

The Observatory brings together faculty members, professionals, students, and administrative staff from diverse disciplinary backgrounds, operating not only as an institutional unit but also as a community of practice that promotes interdisciplinary reflection, collaborative production, and social engagement. Its work addresses animal health and welfare in relation to public health, ecosystem health, biodiversity conservation, food systems, wildlife management, companion animal populations, and the ethical treatment of animals.

One of its main outputs has been the development of semester-oriented bulletins (in total 4 bulletins) conceived as tools for knowledge translation and social appropriation of academic work. These publications combine editorial reflections, data-oriented analyses, expert interviews, dissemination of student and faculty research, policy analysis, and curated resources for academics, professionals, decision-makers, and the broader public. Published issues have addressed topics such as rabies elimination goals, ethical use of animals in education and research, advances in Colombian animal welfare legislation, and the One Health approach.

In addition to the bulletins, the Observatory has promoted academic dialogue through expert interviews, thematic discussions, specialized talks, and academic events that bring together researchers, professionals, students, public institutions, and other stakeholders around issues of national relevance. These activities also contribute to student learning by exposing future professionals to real discussions involving science, ethics, regulation, territorial realities, and public decision-making.

The Observatory further contributes to pedagogical innovation by connecting academic production with critical reflection on socio-environmental and public health challenges. Through dissemination and interdisciplinary dialogue, it promotes systems thinking, ethical analysis, and evidence-informed discussion around biodiversity conservation, environmental sustainability, animal welfare, and public health. Its interdisciplinary structure integrates perspectives from epidemiology, veterinary medicine, environmental sciences, public policy, and social sciences, reinforcing One Health as both an academic framework and a pedagogical approach.

The Observatory has also contributed to policy-oriented discussion through analytical documents and public positions on legislative and regulatory initiatives related to One Health, environmental protection, animal welfare, wildlife management, and animal production. More recently, the development of two dashboards and indicators has expanded its capacity to provide accessible and updated information to support teaching, research, institutional management, and public policy.

Thus, the Observatory operationalizes One Health as an ongoing practice of observation, analysis, communication, and engagement. Within the La Salle model, it represents a key mechanism for translating academic knowledge into public value and for strengthening an informed, interdisciplinary, and context-sensitive approach to health and welfare in Colombia.

#### 2.5.1 Lessons learned and challenges

Despite its establishment as a permanent institutional platform, the Observatory’s operational continuity may be affected by changes in university leadership, institutional priorities, assigned personnel, and available resources. Variations in team composition and formally allocated working time can hinder long-term planning, the continuous updating of information, the consolidation of partnerships, and the preservation of institutional knowledge.

A key challenge is therefore to strengthen the OCSBA’s institutional sustainability through a stable core team, clearly defined responsibilities, recognized workload, adequate financial and technological resources, and effective handover mechanisms. Broader and sustained interdisciplinary participation will also be necessary to consolidate its role as a One Health knowledge translation and policy engagement platform beyond individual administrative periods.

### 2.6 Sixth pillar: experiential community outreach and extension activities: rabies preventive campaigns, AMR capacity-building, UNESCO Chair sustainable development, Peace with Nature Chair and One Welfare certificate

Final-year training activities strengthen the One Health approach by integrating animal, human, and environmental health. Through community-based rabies vaccination and deworming campaigns targeting dogs and cats from shelters and underserved households, students enhance their clinical competencies while promoting responsible pet ownership, animal welfare, disease prevention, and improvements in local environmental and sanitation conditions. In parallel, collaboration with undergraduate students from UniLaSalle Rouen has supported the assessment of knowledge, attitudes, and practices (KAP) related to antimicrobial resistance (AMR) at the One Health interface through surveys administered to veterinarians and final-year students involved in therapeutic decision-making. This initiative identifies gaps between knowledge and implementation, acknowledges constraints within local clinical practice, and reinforces the role of veterinarians as key stakeholders linking clinical care, public health, and responsible antimicrobial use. Ultimately, these activities contribute to the development of a locally adapted antimicrobial stewardship program aligned with international recommendations and grounded in a contextual understanding of animal, human, and environmental health interactions.

To further evaluate the impact of the curriculum on antimicrobial stewardship competencies, structured questionnaires were administered across different stages of the veterinary program. The results demonstrated a progressive improvement in students’ ability to make evidence-based decisions regarding prudent antimicrobial use in small-animal practice. In an internal assessment, students enrolled in the first through sixth semesters correctly identified appropriate antimicrobial use in surgical and hospital-based clinical scenarios in 14.3 and 36.2% of cases, respectively. In contrast, students in the eighth and ninth semesters achieved substantially higher scores, with correct responses in 45.8 and 59.8% of cases, respectively. These differences are likely associated with exposure to curricular content specifically designed to strengthen clinical decision-making and promote responsible antibiotic prescribing. This strategy supports a local antimicrobial stewardship program grounded in a situational assessment aligned with guidance such as PROTEC ME (2024) and ENOVAT (2025). Collectively, these findings suggest that targeted educational interventions enhance antimicrobial stewardship competencies and support the preparation of future veterinarians to address antimicrobial resistance through a One Health framework that recognizes the interconnected impacts of therapeutic decisions on animal, human, and environmental health.

Another key participation was the UNESCO Chair on Sustainable Development together with other institutions like Universidad del Rosario, Universidad Nacional de Colombia, Uniminuto, Swiss Aid, RENAF, OXFAM. Under this collaboration a MOOC course was developed called: SDGs and One Health: Toward a Cross-Cutting and Comprehensive Vision of Healthy Development (“ODS y One Health: hacia la visión transversal e integral del desarrollo saludable”) (80).

This UNESCO chair also highlighted the importance of agroecology in the One Health conversation. The initiative allowed the curriculum to address livestock production systems under the concept of agroecosystems, drawing on agroecological science to design sustainable production systems dependent on soil health as a fundamental subsystem providing water, nutrients, and a rich biota interacting with crops that feed humans and animals—including the principles of “conservation agriculture” (permanent soil cover, minimal tillage, and rotation and diversification of plant species, including trees important for animal welfare). This knowledge was summarized in the book *Conservation Agriculture and Academia* (62).

Likewise, the initiative Peace with Nature Chair at Universidad de La Salle emerged from the 2024 UN Biodiversity Conference (COP16) in Cali, Colombia, as a program fostering *environmental affectivity* (afectividad ambiental) and integral ecology. An interdisciplinary faculty team delivered a student course, complemented by open forums promoting dialogue with environmental organizations. Students collectively developed and presented at COP16 the Manifesto for Socio-Environmental Justice and Peace with Nature, in which One Health is a key conceptual axis alongside bioeconomy, agroecology, and environmental education. These initiatives have expanded to include a faculty seminar and outreach for territorial defenders across Latin America. Currently, with Global Alliance for the Rights of Nature (GARN) and the CLACSO Working Group on Political Ecology(ies) from the South/Abya-Yala, a certificate program called “Rights of Nature and Environmental Peace” links One Health with political ecology (63). This perspective makes it possible both to examine the socio-environmental epidemiologies of extractivism, identifying their differentiated impacts on bodies, communities, animals, and territories and to guide the co-design of transition pathways toward territories of life (61).

Finally, the Certificate Program in Applied Animal Behavior and Welfare has trained 147 professionals from diverse disciplinary backgrounds, led by veterinary medicine and related veterinary sciences (57.8%), biology and biological sciences (12.2%), zootechnics (11.6%), and psychology (4.8%), with the remainder drawn from law, engineering, communication, medicine, humanities, and other fields. As a capstone learning assessment, participants must independently develop and obtain approval of an academic article that integrates and applies the knowledge acquired during the program, either by addressing a specific animal welfare issue or through a systematic review of a selected topic. Certification is therefore contingent upon an individually assessed scholarly output rather than attendance alone. This disciplinary diversity and applied learning approach reflect the spirit of One Welfare, recognizing that animal welfare, human well-being, and environmental stewardship are shared, interdisciplinary responsibilities; an explicit collaborative dimension often overlooked within OH frameworks traditionally centered on disease (64).

#### 2.6.1 Lessons learned and challenges

The experiences described under this pillar show that experiential, community-based learning is an effective vehicle for moving One Health from a conceptual framework to professional practice. Rabies vaccination and deworming campaigns allowed students to connect clinical competencies with animal welfare, responsible ownership, sanitation, and community engagement. The collaboration with UniLaSalle Rouen showed that international student partnerships can generate locally relevant evidence, such as the AMR KAP situational assessment, that informs institutional decision-making. The progressive improvement in prudent antimicrobial use across semesters (from 14.3–36.2% in early semesters to 45.8–59.8% in the eighth and ninth) suggests that deliberately sequenced curricular content can strengthen stewardship competencies. A further lesson is that inter-institutional alliances, including the UNESCO Chair, the Peace with Nature Chair, GARN and CLACSO, broaden One Health beyond its traditional biomedical and disease-centered focus. Through them, agroecology, political ecology, rights of nature, and One Welfare were incorporated, and the alliances produced scalable, open-access outputs such as the MOOC and the COP16 Manifesto.

Several challenges remain. The evidence so far comes from cross-sectional internal assessments. This limits causal attribution to the curriculum and underscores the need for longitudinal, cohort-based evaluation that also follows graduates into professional practice. Similarly, the outcomes of community campaigns (vaccination coverage, changes in household practices, environmental or sanitary improvements) need systematic monitoring to show impact beyond student learning. The One Welfare certificate reached a diverse audience. However, participation from human medicine, law, and the social sciences remains marginal, reflecting the broader difficulty of engaging the human health and social sectors on equal terms.

In Figure 4 the timeline of the 6 pillars regarding the One health integration in Curriculum is presented.

**Figure 4:**
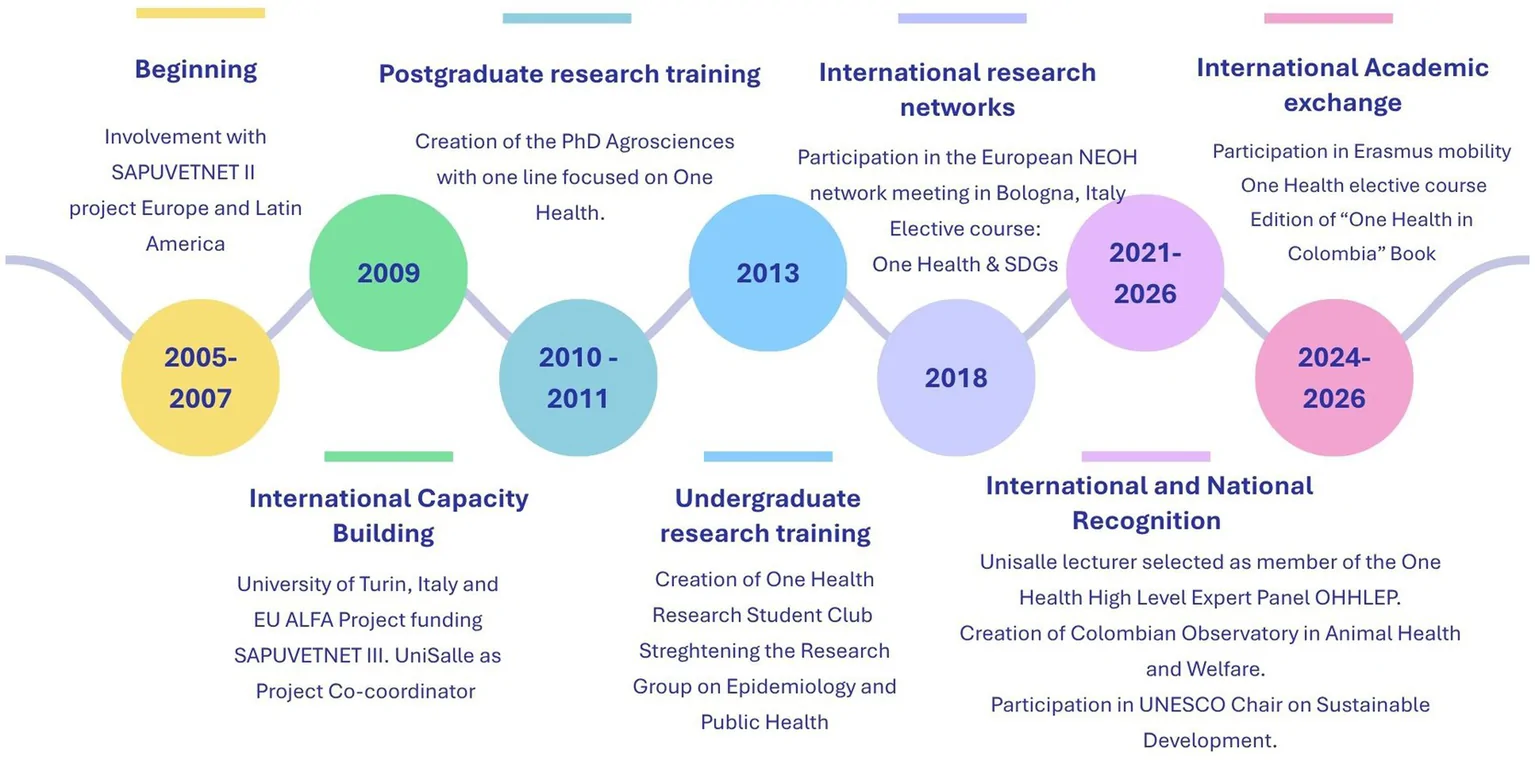
Timeline of the history of One Health integration in the Universidad de La Salle curricula.

## 3 Discussion: lessons learned and practical implications

Several authors have stated that significant challenges must be addressed in the academic training of undergraduate and graduate students, particularly when seeking to integrate the One Health approach across disciplines or, even more so, when implementing it as a dedicated One Health training program (65). Based on the above, various core competencies related to One Health have been proposed, such as communication, ethics, leadership, teamwork, collaboration, and systems thinking (83). This drives the development of strategies to assess the incorporation of One Health competencies into the curriculum, using the model presented as an experience of the Universidad de La Salle, Bogotá. This is grounded in the premise that integrating One Health into the curriculum enables the training of professionals with a high level of socio-environmental awareness, equipped with analytical tools to influence public policies on health, the environment, and animal welfare, among others, and to actively respond to global health and environmental crises (24).

The authors of this study, interviewed two graduates from the veterinary medicine program asking them about the degree of integration of One health during their studies at Universidad de La Salle, Bogota. One of them who studied her postgraduated training with Erasmus mundus joint master degree on Infectious Diseases and One Health, answered with her perspective during her undergraduate studying times: “During my time as a student the One Health approach was taught by some teachers which included it in their lessons, from epidemiology to public health, food security to parasitology, fundamentals of investigation to large animal clinic. From very early in the career, singular teachers made the effort to incorporate a holistic approach to veterinary medicine in order to broaden the narrow concept that the students had. When I started my time at the university, many of us thought that our career was only about small, medium and large animal clinic, nothing more. Along the way we learned that our role as veterinarians goes much further than that. Due to the efforts and mentorship of some teachers, I went on to do a master’s degree in Infectious diseases and One Health. Later, when I went back to the university to present my master’s thesis, I was pleased to see very significant changes regarding the way the university includes the One Health approach into the curriculum. There are specific elective courses on the matter, as well as wild animal medicine and electives which encompass more than production or companion animals and clinics. In the future, I would like to see One Health imbedded in all the classes, from the basic subjects early on in the program, to the clinics, internal medicines, and not only as an elective. In a One Health spirit, the approach should be intertwined in all the curriculum, taught as a way of thinking that leads all of our decisions in the field*”.*

The electives offered during the undergraduate program moved from Conservation Medicine toward ecological, veterinary, epidemiological, and social perspectives shows how the curriculum gradually aligned with the broader One Health and One Welfare paradigms. Literature reviews show that education One Health emphasizes transdisciplinary teaching, integrated pedagogies, and real-world application through case studies, simulations, and field-based learning (66, 67). Likewise wildlife management and wildlife medicine expand One Health thinking beyond disease control by linking biodiversity conservation, ecosystem health, animal welfare, and public health, showing that effective wildlife work has to be interdisciplinary and socially relevant (68, 69). The main challenge is that this integration is still difficult to operationalize because curricula and management systems often remain fragmented, under-resourced, and weakly coordinated across sectors (67). Several sources also show practical gaps in training and implementation, including limited structured exposure to wildlife medicine, insufficient practical experience, low confidence or preparedness, and the need for stronger stakeholder engagement and field-based learning (73). Based on the above, proposing strategies for integration, training and capacity-building together with the official public human and animal health sector is a further task.

Another alumnus shared her experience: “*My education at Universidad de La Salle shaped the way I understand veterinary medicine beyond the individual care of animals. It taught me to recognize the connections among animal health, human health, communities, and the environment. Over time, One Health became more than an academic concept. It became a way of approaching professional decisions and understanding their broader social and environmental implications. This perspective also guides my work as an educator, where I encourage students to see veterinary medicine as a profession that requires technical knowledge, ethical responsibility, and an understanding of the territories and communities in which animals live*”.

As highlighted in this paper and supported by testimonies from veterinary alumni, substantial changes have occurred in the integration of the One Health (OH) approach within the curriculum. The introduction of elective courses, the coordination of diverse student clubs (including graduate and postgraduate students), participation in and development of Communities of Practice (CoP), and the strengthening of networks with national and international institutions, among other advances, reflect a sustained pedagogical effort involving multiple stakeholders. However, several challenges and limitations have emerged during this process and should be addressed in the future:

1. the lack of a thorough transversal integration of the OH approach across all disciplines within the veterinary curriculum;
2. the absence of social-ecological systems (SES) thinking as a core competency in undergraduate and graduate courses;
3. difficulties in integrating social science disciplines (e.g., policy, economics, law, and culture), particularly in agricultural, life, and health sciences undergraduate programmes, which constrains students’ ability to analyse the human behavioural, political, and socio-economic drivers underlying complex health issues;
4. a prevailing view of traditional undergraduate training in which One Health competencies are added on top of subject-specific expertise rather than embedded in ways that transform, but do not replace, specialised, discipline-specific depth;
5. institutional barriers that impede the implementation of a common One Health curriculum across all tertiary faculties related to health, environment, agriculture, engineering, architecture, and social sciences; and
6. still incipient efforts to prioritise transdisciplinary methods that enable students to co-produce solutions with community members, Indigenous knowledge holders, and policy practitioners (70, 71).

One way to strengthen the One Health integration at curriculum level in the Universidad de La Salle, Bogotá is to evaluate the outcomes and initiatives developed over the last decade in order to quantify the impact of basing the curriculum on the ‘One Health’ approach, whereby the number of shared modules, case studies, joint projects and clinical simulations is increased, enabling students’ learning to extend beyond disciplinary boundaries. In this context, companion animals (dogs and cats) have been studied as excellent natural models for translational medicine because they spontaneously develop human diseases (such as cancer and diabetes) and, furthermore, coexist in the same habitat. Studying them accelerates the development of innovative therapies under the One Health approach, benefiting both human and animal health. However, making the most of this requires overcoming barriers to collaboration and terminological differences between doctors and vets through joint interdisciplinary training (66).

The institutionalization of the One Health approach in undergraduate and postgraduate education is constrained by complex financial and structural barriers, a pattern commonly observed in universities such as Universidad de La Salle. Central among these constraints are compartmentalized administrative structures and siloed budgeting frameworks across human health, agricultural, and life science faculties, which encourage competition for resources rather than cross-departmental financial pooling (71, 82). This structural fragmentation is reinforced by conventional academic career metrics and reward systems that create a strong “pull towards the silo,” discouraging transdisciplinary research and curriculum development by treating them as less operationally efficient for institutional advancement (70). Persistent funding asymmetries across disciplines further exacerbate these challenges, as academic capital remains concentrated in disease-centric life sciences, leaving substantial gaps in social sciences, governance, and systems science within degree programs (70, 71). Operational costs constitute an additional obstacle because the delivery of competency-based One Health education requires resource-intensive experiential methods (e.g., field-based immersive learning, problem-based modules, and multi-faculty practical skills training) that demand high initial investment and complex administrative coordination (70). Although these challenges are considerable, they must be overcome through a paradigm shift among institutional leaders and key stakeholders; in this context, strong collaboration among academic partners at the national, regional, and international levels could substantially support progress in this area.

The integration of research incubators *(semilleros*) into the academic training of undergraduate and postgraduate students, based on the One Health approach, represents a necessary pedagogical and curricular strategy, given the need to train professionals capable of addressing complex global issues from a transdisciplinary perspective (84). Thus, the work of research incubators with a One Health approach, developed at Universidad de La Salle University, can serve as a bridge between theory and practice from the early stages of academic training. Furthermore, critical thinking, teamwork and scientific rigour, amongst other skills, are fostered; however, for the One Health approach to be fully integrated into the curriculum, the impacts generated to date must be quantitatively assessed to ensure they lead to a preventive rather than a corrective or curative approach. Furthermore, the academic output generated by the *Research Incubator* has contributed to institutional quality indicators and the accreditation of academic programmes at Universidad de La Salle; in this regard, the One Health model requires cross-cutting reinforcement throughout the Lasallian community. In this regard, we have identified the next contributions to the training process between architecture and agricultural sciences under one health:

The articulation between architecture, One Health and agricultural sciences can be consolidated as a meaningful, interdisciplinary and situated learning experience. The students not only know concepts about rural housing, public health, production or environment; they contrast them with a concrete territory, with situations of vulnerability and with the voices of its inhabitants. This experience favors that learning is built from the relationship between theory, practice, critical reflection and collective action. Rangel Arenas (60) explains that meaningful learning occurs when the new information relates substantively to what the student already knows. In the context of architectural formation, this implies that the concepts of habitat, housing, sustainability, climate adaptation, health and rural production acquire greater meaning when they are connected with the real conditions of San Benito Abad. In this way, students can understand that design decisions have implications for the daily lives, health, productivity, safety and dignity of rural families.

The service-learning approach is equally relevant. The University of La Salle defines service-learning as “a methodology that fosters situational and experiential learning, focused on the application of classroom content through services developed with communities that have real needs” [(79, p. 3), as cited in Rangel Arenas (60, p. 14)]. In academic terms, this definition supports a relationship between training and territory in which university knowledge is put at the service of concrete needs, without reducing communities to passive recipients of assistance.

Experience also strengthens communication, argumentative and interdisciplinary interaction skills. Rangel Arenas (77) points out that the spaces of conversation and socialization enrich the training process, helping students overcome “the fear of public speaking” and strengthen “their communication skills” (p. 8). This contribution is significant for complex experiences like One Health, in which students must learn to communicate findings and proposals to diverse audiences: rural communities, teachers, professionals from different disciplines, public institutions and social organizations.

### 3.1 Generalisability

The six pillars described here were not built simultaneously, and institutions with fewer resources need not replicate them as a package. Our experience suggests a phased pathway. A first, low-cost phase can rest on two entry points: a research seedbed anchored in an existing research group, and a single One Health elective open to students from several programmes. Both can draw on freely available materials, such as the open-access SAPUVETNET teaching manual, the MOOC “SDGs and One Health”, or serious games like the One Health Mural. A second phase can reframe activities that already exist, rather than create new ones. One Health content can be made explicit in core courses such as epidemiology, public health, parasitology and clinics, and in routine community activities such as vaccination campaigns, where students can connect clinical tasks with animal welfare, sanitation and community engagement. At this stage, a baseline curriculum self-assessment (72) can identify where competencies are already taught and where gaps remain. A third phase can extend the model through partnerships with national and regional networks, virtual exchanges and competitive mobility calls, which require institutional agreements more than dedicated budgets. Finally, a knowledge translation platform can begin modestly, for example as a student-led semester bulletin, before being formalised as an observatory with a stable team. Across all phases, two conditions appear more decisive than funding: at least one committed faculty champion supported by a co-lead to ensure succession, and institutional recognition of One Health work in faculty workloads. Other features of our case are context-dependent and should not be seen as prerequisites. These include EU-funded networks, an A1-ranked research group and the visibility provided by hosting COP16 at country level.

## 4 Conclusion

Since 2009, Universidad de La Salle has progressively moved One Health in the Faculty of Agricultural Sciences from an externally introduced concept towards an institutional practice. Rather than relying on a single course or centre, the university linked international cooperation, research, undergraduate inquiry, curricular innovation, knowledge translation and community engagement across levels of training. The resulting model is multi-level and grounded in the realities of Colombian rural and peri-urban life, but it remains a process under construction rather than a finished achievement, and its lessons and challenges are as relevant to other institutions as its results.

Early and sustained engagement with the SAPUVETNET network, later extended through Erasmus+ partnerships with the University of Turin and UniLaSalle Rouen, shows that international cooperation can act as a catalyst for institutional change rather than mere academic exchange. It left installed capacities, including research lines, postgraduate programmes and open educational materials, that outlasted external funding. The key lesson is that sustainability depends less on external resources than on anchoring initiatives in curricular structures and in *a prior* institutional vision, such as Agrosciences and the “new rurality.” However, this trajectory has relied on a small number of institutional champions, which raises concerns about generational succession, and its materials need updating to reflect current frameworks such as the One Health Joint Plan of Action and global AMR and climate adaptation plans.

The Epidemiology and Public Health Research Group has anchored One Health claims in local data on zoonoses, AMR, vector-borne diseases and the socio-environmental determinants of health, and has increasingly incorporated gender, care work and climate resilience through participatory approaches. The main pending task is to translate this evidence into governance. Colombia still lacks a national One Health Joint Plan of Action and a coordinated university network providing technical advice, and the Faculty has yet to develop a training portfolio for emerging areas such as agricultural emergency and disaster risk management. Promoting these instruments, in a context of declining public health funding, is a strategic priority.

In workforce training, the combination of research seedbeds, interdisciplinary and veterinary electives, postgraduate programmes and community-based activities offers students repeated opportunities to engage with the human–animal–environment interface. Internal assessments suggest that this exposure is associated with a broader and more systemic understanding of One Health and with more prudent antimicrobial decision-making in later semesters. Semilleros and electives have proven to be effective, low-cost entry points, and experiential, community-based learning helps move One Health from concept to practice. Nevertheless, participation remains predominantly veterinary, some formative spaces such as the Conservation Medicine electives have been discontinued, and the available evidence is cross-sectional and internal. It does not yet capture changes in competencies over time, community outcomes or graduates’ professional practice.

The Colombian Observatory of Animal Health and Welfare and inter-institutional alliances with the UNESCO Chair, the Peace with Nature Chair, GARN and CLACSO have broadened One Health beyond its disease-centred focus, incorporating One Welfare, agroecology, political ecology and the rights of nature, and generating open outputs such as the MOOC and the COP16 Manifesto. These platforms depend, however, on changes in leadership, staffing, recognised workload and resources, and on partnerships that are often time-bound. Their continuity will require stable core teams, handover mechanisms and formal institutional support.

Across the six pillars, a shared challenge emerges: moving from initiatives sustained by individual leadership towards formal institutionalisation within curricula, faculty workloads and budgets. This includes embedding One Health transversally across all courses rather than only in electives, as students and alumni themselves request. It also involves strengthening social-ecological systems thinking and engaging human health, environmental and social sciences on equal terms, while overcoming administrative and funding structures that pull towards disciplinary silos.

Building on this trajectory, the next step is to move from documenting initiatives to systematically assessing the degree to which One Health competencies are embedded across the curriculum. To this end, the Faculty will apply a structured curriculum self-assessment such as the One Health Curriculum Self-Assessment Checklist developed by the University College Dublin One Health Centre (72). This checklist evaluates integration across six domains using a four-level rubric applied to curriculum content, learning activities and assessment strategies. It would provide a baseline for guiding transversal integration and could be complemented by longitudinal, cohort-based evaluation that follows graduates into professional practice and by systematic monitoring of community-level outcomes.

Taken together, this experience illustrates how a Latin American university, without large dedicated funding streams, can build an internationally connected and locally grounded One Health education ecosystem. It also shows the limits that such an ecosystem must address to become durable. By documenting both achievements and pending challenges, Universidad de La Salle offers other resource-constrained institutions in the region a transferable roadmap: one that treats One Health not as isolated content, but as a sustained institutional commitment to generating evidence, training a new generation of professionals and embedding systems thinking in health education.

## Data Availability

The original contributions presented in the study are included in the article/supplementary material, further inquiries can be directed to the corresponding author.
